# Human induced pluripotent stem cell-derived neural stem/progenitor cell therapy for spinal cord injury: preclinical advances and translational perspectives

**DOI:** 10.1093/stcltm/szaf073

**Published:** 2026-01-09

**Authors:** Ryo Ogaki, Narihito Nagoshi, Hideyuki Okano, Masaya Nakamura

**Affiliations:** Department of Orthopaedic Surgery, Keio University School of Medicine, Shinjuku-ku, Tokyo 160-8582, Japan; Department of Orthopaedic Surgery, Keio University School of Medicine, Shinjuku-ku, Tokyo 160-8582, Japan; Keio University Regenerative Medicine Research Center, Kawasaki, Kanagawa 210-0821, Japan; Department of Orthopaedic Surgery, Keio University School of Medicine, Shinjuku-ku, Tokyo 160-8582, Japan

**Keywords:** spinal cord injury, induced pluripotent stem cells (iPSCs), stem/progenitor cell, cell transplantation, progenitor cells, pluripotent stem cells, clinical translation

## Abstract

Spinal cord injury (SCI) causes irreversible neurological damage and remains a major clinical challenge due to the lack of effective regenerative therapies. Human-induced pluripotent stem cells (hiPSCs) and their derivatives, hiPSC-derived neural stem/progenitor cells (hiPSC-NS/PCs), have demonstrated potential to promote neural repair and functional recovery. The world’s first clinical trial using hiPSC-NS/PCs in the subacute phase of SCI has already been initiated. In contrast, chronic SCI—despite accounting for the majority of clinical cases—remains difficult to treat due to pathological barriers such as widespread demyelination, cavitation, scar formation, and persistent inflammation. Recent efforts to overcome these obstacles include combinatorial strategies incorporating rehabilitation, biomaterial scaffolds, pharmacological adjuvants, and robotic-assisted therapy as well as gliogenic or regionally patterned hiPSC-NS/PCs. Preclinical models have demonstrated that such multifaceted approaches can enhance graft survival, axonal regeneration, and functional recovery. In this review, we provide an overview of the biological characteristics, mechanisms of action, and recent advances in preclinical and clinical research on hiPSC-NS/PCs transplantation for SCI. We also discuss future perspectives and challenges toward clinical application. Collectively, these efforts underscore the diverse, innovative, and translational potential of hiPSC-based regenerative medicine for SCI.

## Graphical abstract

**Graphical Abstract:**
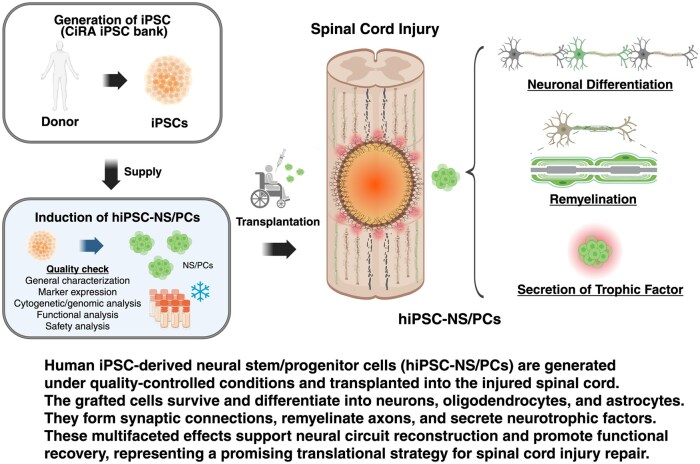

Significance statement Spinal cord injury (SCI) remains a debilitating neurological condition with few effective treatment options. Human-induced pluripotent stem cell–derived neural stem/progenitor cells (hiPSC-NS/PCs) offer a promising regenerative strategy by enabling neural replacement, promoting remyelination, and modulating the injured microenvironment. Progress in preclinical models, together with the initiation of the first clinical trial of hiPSC-based therapy for SCI, has further strengthened the translational potential of this approach. This concise review synthesizes current evidence on the biological properties, mechanisms of action, and safety considerations of hiPSC-NS/PCs and outlines key challenges and opportunities for advancing clinical application.

## Introduction

### Epidemiology and therapeutic challenges of spinal cord injury

Spinal cord injury (SCI) is a sudden-onset condition that results in severe neurological dysfunction. It often affects previously healthy individuals who abruptly fall into a state of profound paralysis, and the potential for spontaneous recovery remains extremely low. Globally, more than 15 million people are affected by SCI, with most cases resulting from traumatic causes such as falls, motor vehicle accidents, sports injuries, or violence.^1^ In addition to traumatic causes, SCI can also result from nontraumatic conditions such as degenerative diseases, vascular disorders, or tumors. In recent years, with the progression of global aging, the prevalence of spinal canal stenosis and other degenerative spinal conditions has increased. As a result, even minor trauma—such as falls on level ground—can cause SCI, particularly cervical SCI, in elderly individuals.^2^ Loss of independence and reduced work capacity due to SCI impose serious socioeconomic burdens not only on the affected individuals but also on their families and support systems. Given this background, SCI is expected to become an increasingly significant condition in terms of both medical and social impact. Since the establishment of induced pluripotent stem cell (iPSC) technology,^3^^,^^4^ we have focused our research on applying human iPSCs (hiPSCs) to regenerative therapy for SCI. In this review, we summarize recent advances in the treatment of SCI using hiPSC-derived neural stem/progenitor cells (hiPSC-NS/PCs), focusing on the evaluation of their efficacy and safety in both preclinical and early clinical studies.

### Potential of stem cell and iPSC-based therapies for SCI

In addition to the irreversible destruction of neural tissue caused by the primary insult, SCI also involves secondary processes such as inflammation, axonal degeneration, demyelination, and glial scar formation. These pathological changes significantly hinder the reconstruction of neural circuits, making spontaneous functional recovery extremely limited.^5^ Current standard treatments aim to limit the progression of damage and promote compensatory functional recovery through rehabilitation. However, no existing therapy can regenerate or repair the damaged neural tissue itself.

In this context, stem cell–based therapy has emerged as a promising approach within the field of regenerative medicine. Stem cells possess both self-renewal capacity and multipotency and are expected to contribute to cell replacement at the injury site, reconstruction of neural circuits, remyelination, neuroprotection, and modulation of inflammation. More recently, the advent of iPSC-technology has accelerated this research by providing an ethically acceptable and immunologically favorable source of cells that can be expanded indefinitely.^3^^,^^4^ iPSCs can be generated from a patient’s own somatic cells, offering a major advantage for personalized medicine. Thus, stem cell therapy—particularly using iPSCs—has attracted increasing attention as a potential fundamental treatment for SCI, and basic and preclinical studies toward clinical application are actively progressing worldwide.^6^^,^^7^

### Characteristics and clinical applications of neural progenitor cells by origin

Among stem cell–based therapies, neural stem/progenitor cells (NS/PCs) have attracted particular attention in SCI. This is due to their strong differentiation potential into central nervous system (CNS) lineages and responsiveness to the local microenvironment.^8^^,^^9^ NS/PCs from different sources have been studied in experimental and clinical settings. These include human fetal tissue–derived NS/PCs, human embryonic stem cells (hESCs)–derived neural lineages such as oligodendrocyte progenitor cells (OPCs), and human iPSC–derived NS/PCs. Each source exhibits distinct characteristics in terms of biological properties, differentiation potential, safety profile, ethical considerations, and supply stability, resulting in differing degrees of clinical applicability.

#### Human fetal–derived NS/PCs

NS/PCs derived from human fetal CNS tissue are obtained from cells at physiological developmental stages, exhibiting intrinsic neurogenic potential and excellent adaptability to the host CNS environment. Preclinical studies using animal models of SCI have reported successful reconstruction of neural circuits and improved motor function, and clinical trials involving transplantation of human fetal-derived NS/PCs into human patients have also been conducted.^10–12^ A first-in-human Phase I clinical trial evaluated the safety and feasibility of transplanting NSI-566, a fetal spinal cord–derived NS/PC line, into patients with chronic thoracic SCI. The procedure was well tolerated in all four subjects, with no serious adverse events, and preliminary improvements in neurological function were reported. A 5-year follow-up study further confirmed the long-term safety of the procedure and showed sustained improvements in motor and sensory scores, as well as electrophysiological evidence of functional recovery.^11^^,^^12^ However, ethical concerns regarding the use of human fetal tissues, donor dependency, and instability of cell supply remain significant limitations in clinical applications, posing challenges for widespread adoption as a generalized therapeutic strategy.

#### Human ESC–derived OPCs

hESCs are a pluripotent cell source with unlimited self-renewal capacity.^13^ hESCs can be efficiently directed toward specific neural lineages, including OPCs, under defined conditions, making them favorable for standardization and scalable product manufacturing.^14–16^ Preclinical studies demonstrated that transplantation of hESC-derived OPCs promoted remyelination and motor recovery in rodent models of subacute SCI, although no benefit was observed in chronic models.^15^ Building on these results, a first-in-human trial using hESC-derived OPCs (LCTOPC1; previously known as GRNOPC1 and AST-OPC1) in patients with acute thoracic SCI confirmed safety and feasibility. A 10-year follow-up study reported no tumor formation, lesion enlargement, or neurological deterioration, supporting the long-term safety of hESC-derived OPC therapy in humans.^16^ Building on these findings, hESC-derived OPCs were evaluated in a Phase 1/2a trial for subacute cervical SCI (AIS-A, C4–C7).^17^ In this study, 96% of participants regained at least one motor level on one or both sides, and 32% recovered two or more levels, with no unexpected adverse events. However, this degree of improvement could partly reflect natural progression or spontaneous recovery, and causal relationships to cell therapy should be interpreted with caution. In addition, a subsequent clinical trial has been initiated in 2025 and is currently ongoing. The results of this study are awaited to further clarify the safety and therapeutic potential of this approach.

In Japan, the regulatory environment now permits the clinical application of hESC-derived products under defined quality and safety standards. For instance, clinical studies applying hESC-based therapies have been initiated for congenital metabolic disorders.^18^ However, broader social and ethical consensus on the clinical use of hESCs remains under development. Moreover, the risks of immune rejection, karyotypic instability, and limited donor availability continue to warrant careful evaluation in translational applications.^8^^,^^19^

#### Human iPSC–derived NS/PCs

**Figure 1.**
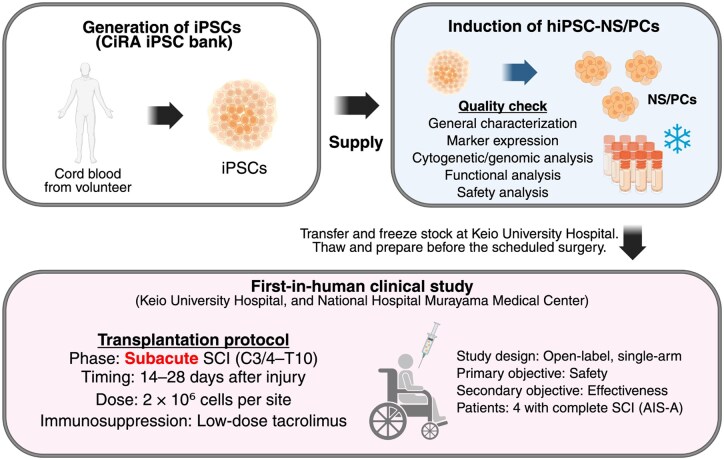
hiPSC-NS/PC preparation and first-in-human transplantation for subacute SCI. Somatic cells derived from umbilical cord blood of healthy donors were reprogrammed into induced pluripotent stem cells (iPSCs) and differentiated into neural stem/progenitor cells (NS/PCs) under defined conditions. The resulting human iPSC-derived NS/PCs (hiPSC-NS/PCs) underwent comprehensive quality control, including general characterization, marker expression, cytogenetic/genomic analysis, functional analysis, and safety evaluation, before cryopreservation. The schematic also summarizes the first-in-human clinical study, in which four patients with complete subacute SCI (AIS-A, C3/4–T10) received 2 × 10^6^ cells per lesion site within 14–28 days after injury, with transient low-dose tacrolimus as immunosuppression.

hiPSC-NS/PCs possess pluripotency comparable to that of hESCs, while presenting fewer ethical concerns. Moreover, as they can be generated from the patient’s own somatic cells, they offer significant advantages in terms of personalized medicine and reduced risk of immune rejection. Nevertheless, concerns remain regarding genomic instability and off-target differentiation, which require careful monitoring. Currently, strategies to mitigate tumorigenic risks—such as the elimination of undifferentiated cells and rigorous quality control of transplanted cells—are being actively developed, and clinical application of hiPSC-based therapy for SCI is steadily progressing. In Japan, the first clinical study using donor-derived hiPSC-NS/PCs for subacute complete SCI (AIS-A) has been conducted^20^; this is summarized in Figure 1. In this trial, four patients with injuries ranging from C3/4 to T10 received cell transplantation within 28 days of injury. The first transplantation was performed in 2021, and the observation of the patients was completed in 2024. At the time of writing, detailed safety and functional outcomes from this study have not been published and therefore are not included in this review. This trial represents a critical milestone toward the clinical application of hiPSC-NS/PCs transplantation for SCI. Future directions include extending the approach to chronic SCI and optimizing therapeutic efficacy through combination strategies, such as rehabilitation and pharmacological interventions.

Major clinical trials of NS/PCs and OPCs transplantation therapy for SCI reported to date have been classified by cell source^10–12^^,^^16^^,^^17^^,^^21–24^ (Table 1).

**Table 1.** Clinical trials of stem cell–based therapies for SCI.

	Start year	Cell source	Developer	Target stage (level)	Phase	Notes
**Fetal-derived**	2011	Fetal brain–derived NSCs (HuCNS-SC)	StemCells Inc.	Subacute (thoracic)	I/II	Safe; limited sensory/motor improvement.
	2014	Fetal brain–derived NSCs (HuCNS-SC)	StemCells Inc.	Chronic (cervical)	II	Early trend not sustained; terminated for futility.
	2014	Fetal spinal cord–derived NSCs (NSI-566)	Neuralstem	Chronic (thoracic)	I	Safe; 3 of 4 subjects showed improvement.
	2017	Fetal spinal cord–derived NSCs (NSI-566)	Neuralstem	Chronic (cervical)	N/A (planned)	IRB-approved cervical expansion; results unpublished as of 2025.
	2015	Fetal brain–derived NS/PCs	Yonsei Univ.	Acute–Chronic (cervical)	I/IIa	Safe; AIS improvement in 5/19; comparative study with rehabilitation-only group.
**ESC-derived**	2010	Human ESC–derived OPCs (GRNOPC1)	Geron	Acute	I	First-ever hESC-based SCI trial; stopped due to company reprioritization.
	2015	Human ESC–derived OPCs (AST-OPC1)	Asterias → Lineage	Subacute (cervical)	I/IIa	Safe; 96% showed motor gain; no tumors.
	2025	Human ESC–derived OPCs (LCTOPC1)	Lineage	Subacute/Chronic	I	Includes chronic SCI; novel injection method tested. (ongoing)
	2021	Human ESC–derived NPCs (PSA-NCAM+)	S.Biomedics	Subacute (cervical)	I/II	Intrathecal (not intraparenchymal) injection; safety/efficacy under evaluation. (ongoing)
**iPSC-derived**	2020	Human iPSC–derived NS/PCs	Keio Univ.	Subacute	I	Safety/efficacy under evaluation; first hiPSC-NS/PC trial.

Clinical trials are listed by start year and summarize key information on the cell source (fetal-, ESC-, or iPSC-derived), developer, target stage and level of SCI, clinical phase, and notable outcomes.

Abbreviations: AIS, American Spinal Injury Association Impairment Scale; ESC, embryonic stem cell; iPSC, induced pluripotent stem cell; NS/PCs, neural stem/progenitor cells; OPCs, oligodendrocyte progenitor cells; IRB, institutional review board. LCTOPC1 was previously referred to as GRNOPC1 and AST-OPC1.

## hiPSC-NS/PCs transplantation therapy for SCI

### Mechanisms of hiPSC-NS/PCs transplantation

Transplantation therapy using hiPSC-NS/PCs is considered a promising fundamental treatment for SCI. Our studies have shown that transplanted hiPSC-NS/PCs can survive and may contribute to both functional improvement and tissue preservation of the injured spinal cord, without tumor formation being observed in those experiments, in both rodent and non-human primate SCI models^25–41^ (Table 2). The therapeutic mechanisms are mainly attributed to the following three aspects.

**Table 2.** Therapeutic preclinical studies of hiPSC-derived NS/PCs transplantation for SCI.

Author, year	Phase	hiPSC	Motor score	Safety
**Nori et al.^25^**	Subacute	201B7	Improved vs PBS	No tumorigenicity; low proliferation
**Kobayashi et al.^26^** **– Marmoset study**	Subacute	201B7	Improved vs PBS	No tumorigenicity; low proliferation
**Okubo et al.^27^** **– GSI pretreatment**	Subacute	253G1, 836B3 (tumorigenic), 201B7	Improved vs PBS and control; prevented deterioration in tumorigenic clones	No tumorigenicity; proliferation reduced
**Okubo et al.^28^** **– GSI pretreatment**	Chronic	201B7, 414C2	Modest improvement vs PBS	No tumorigenicity; proliferation reduced
**Kojima et al.^29^** **– HSVtk suicide gene**	Subacute	253G1 (tumorigenic)	Recovery maintained in HSVtk group; deterioration in control	No tumorigenicity; proliferative cells ablated
**Kajikawa et al.^30^** **– Region-specific NPCs**	Subacute	201B7, 414C2	Spinal cord-type NPCs improved vs PBS; Forebrain-type no effect	No tumorigenicity
**Kamata et al.^31^** **– Gliogenic NS/PCs**	Subacute	WJ14s01	Improved vs PBS	No tumorigenicity; low proliferation
**Ito et al.^32^** **– LOTUS overexpression**	Subacute	414C2	Improved vs control TP	No tumorigenicity; low proliferation
**Kawai et al.^33^** **– Chemogenetic stimulation (DREADD)**	Subacute	414C2	Improved vs control TP	No tumorigenicity; low proliferation
**Kitagawa et al.^34^** **– DREADD inhibition**	Subacute	414C2	Neuronal inhibition reduced recovery	No tumorigenicity; low proliferation
**Shibata et al.^35^** **– C5aRA adjunct**	Acute	414C2	C5aRA+TP improved vs PBS and vs TP only	No tumorigenicity; low proliferation
**Shibata et al.^36^** **– Transplantation + rehabilitation**	Chronic	YZWJs513	Modest improvement vs control TP	No tumorigenicity; low proliferation
**Hashimoto et al.^37^** **– HGF-releasing scaffold + TP**	Chronic	YZWJs513	Improved vs control TP	No tumorigenicity; low proliferation
**Suematsu et al.^38^** **– HGF pretreatment**	Subacute	YZWJs513	Improved vs TP and HGF alone	No tumorigenicity; low proliferation
**Yoshida et al.^39^** **– TP + rehabilitation + Sema3Ai**	Chronic	YZWJs513	Modest improvement vs TP + rehabilitation	No tumorigenicity; low proliferation
**Saijo et al.^40^** **– CPTX overexpression**	Subacute	YZWJs513	Modest improvement vs control TP	No tumorigenicity; low proliferation
**Ito et al.^41^** **– Scar resection + dECM scaffold**	Chronic	QHJI01s04	No functional recovery	No tumorigenicity

This table summarizes therapeutic preclinical studies conducted in our laboratory, indicating the experimental phase, human iPSC (hiPSC) line, efficacy outcomes (motor function improvement compared with controls), and safety findings (tumorigenicity and cell proliferation).

Abbreviations: C5aRA, C5a receptor antagonist; CPTX, cerebellin-1/pentraxin-1 chimera; dECM, decellularized extracellular matrix; DREADD, designer receptor exclusively activated by designer drug; GSI, γ-secretase inhibitor; HGF, hepatocyte growth factor; HSVtk, herpes simplex virus type 1 thymidine kinase; LOTUS, lateral olfactory tract usher substance; Sema3Ai, semaphorin 3A inhibitor.

First, hiPSC-NS/PCs differentiate into neurons and form synaptic connections with host circuits. Chemogenetic manipulation using DREADD (Designer Receptors Exclusively Activated by Designer Drugs) has demonstrated that neuronal activity of graft-derived cells causally contributes to motor function recovery.^33^^,^^34^ Inhibition of graft activity led to temporary motor decline, while enhancing activity promoted synapse formation, prevented atrophy, and improved locomotor function without inducing pain. These findings are further supported by studies showing that hiPSC-NS/PCs extended long-distance axons and formed synapses with host neurons in a rat SCI model.^42^ More recently, optogenetic stimulation combined with calcium imaging has demonstrated that graft-derived neurons can form functional synaptic networks with host circuits after SCI.^43^ In addition, fetal-derived NS/PCs integrate into host circuits and project widely throughout the CNS in a nonhuman primate model.^44^

Second, hiPSC-NS/PCs can differentiate into oligodendrocytes, which contribute to remyelination and restoration of axonal conduction.^45^^,^^46^ We previously developed a hiPSC-NS/PC line with a strong propensity for glial differentiation and demonstrated that when transplanted into a subacute SCI model, approximately 40% of the grafted cells differentiated into oligodendrocytes.^31^^,^^47^ This represents a substantial improvement over the ∼3% differentiation rate observed in the original hiPSC-NS/PCs. These cells promoted functional recovery by remyelinating host axons, indicating that hiPSC-NS/PCs can contribute to both structural and functional reconstruction of the CNS beyond neuronal replacement. Similar approaches have been reported, showing that oligodendrogenic human neural precursor cells promoted widespread remyelination, tissue sparing, and motor recovery after SCI without tumor formation in preclinical models.^48^ In particular, transplantation of these cells in a subacute SCI model led to efficient differentiation into mature oligodendrocytes, robust remyelination of host axons, and preservation of white matter, resulting in significant functional recovery without evidence of tumorigenicity.

Third, transplanted hiPSC-NS/PCs secrete neurotrophic factors and cytokines that mitigate secondary injury and modulate the host environment. Even in their immature state, they can exert neuroprotective and anti-inflammatory effects, reducing gliosis and supporting repair. In vivo analyses have shown upregulation of factors such as ciliary neurotrophic factor and insulin-like growth factor-1, with additional stimulation enhancing brain-derived neurotrophic factor (BDNF), glial cell line–derived neurotrophic factor (GDNF), and neurotrophin-3 (NT-3), underscoring their paracrine potential to promote survival, axonal regeneration, and white matter preservation.^49^ RNA-seq studies further revealed that the secretory profile of grafted NS/PCs is highly environment-dependent, differing from in vitro predictions.^50^ Moreover, specific factors like GDNF can modulate Notch signaling, redirecting graft fate toward neurons, promoting tissue sparing, and enhancing integration.^51^ Collectively, these findings highlight the multifaceted trophic support of NS/PCs, which contributes significantly to tissue preservation and functional recovery.

### Subacute phase of SCI: therapeutic window and combination strategies

To maximize the multifaceted therapeutic effects of hiPSC-NS/PCs transplantation, the timing of intervention is critical. The acute phase is characterized by intense inflammatory responses, which can hinder graft survival and differentiation. In contrast, the chronic phase presents a hostile environment for engraftment due to the accumulation of glial scarring and axonal growth inhibitors.^52^ Therefore, the subacute phase is considered the optimal window for transplantation. This time frame has been adopted in a clinical trial led by Keio University (jRCTa031190228).^20^

In recent years, increasing attention has been directed toward combination strategies aimed at enhancing the efficacy of cell transplantation in subacute SCI. Hepatocyte growth factor (HGF), known for its anti-inflammatory, angiogenic, and neuroprotective properties, has been shown to improve the injured spinal cord environment, primarily in acute SCI models.^53–57^ In particular, a recent preclinical study administered recombinant human HGF during the acute phase and subsequently transplanted hiPSC-NS/PCs in the subacute phase.^38^ This sequential approach enhanced graft survival, promoted remyelination and neuronal regeneration, and led to greater motor recovery compared to either treatment alone. These findings indicate that, in this context, HGF may serve as a preconditioning strategy that facilitates a more permissive microenvironment for subsequent transplantation in the subacute phase. Moreover, another strategy involving hiPSC-NS/PCs engineered to express CPTX (a synthetic excitatory synapse organizer composed of Cbln1 and pentraxin domains) demonstrated a robust increase in excitatory synapse formation at the graft site.^40^ These findings collectively suggest that combining cell transplantation with molecular or genetic modifications represents a promising and multifaceted therapeutic strategy for SCI.

### Safety and tumorigenicity control in hiPSC-NS/PCs transplantation

hiPSC-NS/PCs are a promising cell source for regenerative therapy in SCI. However, tumorigenicity remains a significant concern in clinical applications.^58^ Even when hiPSCs are confirmed to be safe at the time of establishment, some cells may exhibit tumor-like growth or resistance to differentiation after being induced into NS/PCs and transplanted.^59^ Strategies to eliminate or control tumorigenic cells are essential to mitigate these risks.

One of the primary approaches is to select hiPSC-NS/PC lines with verified safety. Comparative studies between tumorigenic and nontumorigenic lines have revealed that tumor suppressor genes are hypermethylated in tumorigenic NS/PCs.^60^ Moreover, even initially validated lines showed increased tumorigenic potential after multiple passages due to accumulated epigenetic abnormalities.

To further suppress tumorigenic cells, a pretreatment strategy targeting Notch signaling using a γ-secretase inhibitor (GSI) has been developed.^27^^,^^28^ A short-term, one-day treatment with GSI promoted neuronal differentiation and reduced proliferation and expression of tumor-associated genes in hiPSC-NS/PCs. Transplantation of these cells into an SCI model resulted in mature neuron formation without tumor development, in contrast to non-treated controls.

To ensure the safety and consistency of transplanted cells, rigorous quality control procedures have been established. These include flow cytometric confirmation of negative expression for undifferentiated markers such as OCT4 and TRA-1-60, as well as assessments of cell morphology, expression of neural and glial markers, karyotype stability, and differentiation and functional capabilities.^20^ These measures are integrated into the cell selection process for current clinical use.

Through the integration of careful cell line selection, molecular control strategies, and multilayered quality assessments, the risk of tumorigenicity associated with hiPSC-NS/PCs transplantation is being steadily reduced. Establishing long-term post-transplant monitoring systems will be key to ensuring further safety and advancing clinical application.

## hiPSC-NS/PCs transplantation for chronic SCI

### Challenges and limitations of hiPSC-NS/PCs transplantation in chronic SCI

**Figure 2.**
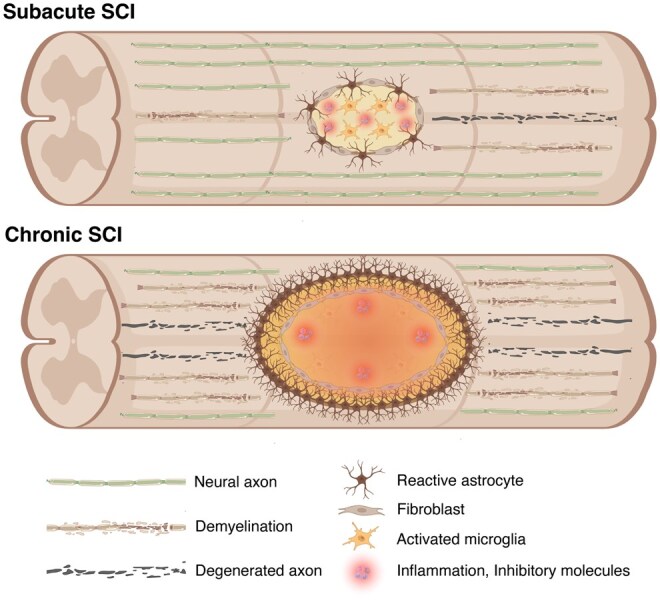
Pathological features of subacute and chronic phases after SCI. The schematic illustrates pathological features of different phases after SCI. In the subacute phase, axonal degeneration and demyelination occur together with reactive astrocytes, activated microglia, fibroblasts, and persistent inflammation with inhibitory molecules. In the chronic phase, cavity formation surrounded by a dense astrocytic and fibrotic scar, continued axonal degeneration, and a sustained inflammatory environment with inhibitory molecules create a nonpermissive microenvironment that limits axonal regeneration and functional recovery.

While therapeutic strategies using hiPSC-NS/PCs transplantation have made progress in the acute and subacute phases of SCI, effective treatments for chronic SCI—which accounts for approximately 95% of all SCI cases—remain largely undeveloped and represent a major clinical challenge.^2^^,^^61^ In the subacute phase, the injury environment is relatively permissive, enabling better graft survival, integration, and safety, whereas in the chronic phase, scarring, cavitation, and demyelination create inhibitory conditions that limit functional outcomes and can affect graft behavior^62–64^ (Figure 2). Preclinical studies have demonstrated that functional recovery is markedly greater when transplantation is performed in the subacute phase, while chronic transplantation is restricted by the inhibitory microenvironment.^52^

In response to this clinical need, clinical trials using fetal brain- or spinal cord–derived NS/PCs have been initiated in the United States.^11^^,^^12^^,^^22^ However, fetal tissue–derived cells raise ethical concerns and suffer from limited supply. Therefore, hiPSC-NS/PCs have emerged as a promising alternative cell source, offering both stable availability and reduced ethical constraints. Nevertheless, preclinical studies to date have not demonstrated sufficient efficacy of NS/PCs transplantation alone in the chronic phase of SCI, highlighting the need to develop more effective and sustainable therapeutic strategies.

To address this challenge, we previously investigated the transplantation of hiPSC-NS/PCs pretreated with GSI, aimed at suppressing undifferentiated potential and enhancing neuronal differentiation, using a mouse model of chronic SCI.^28^ While this approach demonstrated safety and some therapeutic efficacy, the observed functional improvements were limited. Therefore, we have continued to explore a range of additional strategies to further enhance treatment outcomes in chronic SCI.

### Experimental approaches to hiPSC-NS/PCs transplantation in chronic SCI

To enhance the therapeutic efficacy of hiPSC-NS/PCs transplantation for chronic SCI, combination therapies with rehabilitation have been investigated.^65^ In a chronic SCI mouse model, quadrupedal treadmill training based on the principle of overload was applied after hiPSC-NS/PCs transplantation. This approach improved graft survival, promoted neuronal differentiation, and expanded the area of synapse formation and serotonergic fiber regeneration around the lesion site. Locomotor improvements were positively correlated with running distance and training speed and were also associated with increased expression of neurotrophic factors such as BDNF and NT-3.^36^ These findings suggest that combining hiPSC-NS/PCs transplantation with rehabilitation can promote neural circuit regeneration and maturation, thereby enhancing functional recovery in chronic SCI.

Chondroitinase ABC (C-ABC) enzymatically digests chondroitin sulfate proteoglycans in the glial scar, thereby alleviating inhibitory signaling after SCI. Its efficacy has been demonstrated in acute and subacute rodent models and in nonhuman primates, where it promoted corticospinal sprouting, synapse formation, and functional recovery.^66^^,^^67^ In chronic SCI, C-ABC combined with treadmill rehabilitation enhanced serotonergic and corticospinal sprouting and improved locomotor function.^68^ Moreover, C-ABC pretreatment facilitated subsequent hiPSC-NS/PCs transplantation,^69^ while sustained-release C-ABC in combination with oligodendrogenic neural progenitor cells further promoted graft survival, oligodendrocyte differentiation, remyelination, and functional recovery.^70^ In addition to C-ABC, Semaphorin 3A inhibitors, which independently promote axonal regeneration, showed synergistic effects when combined with rehabilitation.^68^ When further combined with hiPSC-NS/PCs transplantation, this triple therapy enhanced neuronal and oligodendrocyte differentiation, axonal regeneration, and locomotor recovery.^39^

For more severe injuries such as complete spinal cord transection, a therapeutic strategy using collagen scaffolds that gradually release HGF has been developed.^37^ This approach promoted angiogenesis, suppressed inflammation, and reduced scar and cavity formation, resulting in improved locomotor and bladder functions. More recently, a triple therapy involving scar tissue removal, a decellularized extracellular matrix scaffold derived from kidney tissue, and hiPSC-NS/PCs transplantation has also been attempted.^41^ This strategy led to improved graft survival, enhanced axonal regeneration, and reduced cavity formation, demonstrating notable structural repair even in chronic SCI.

Our group has also focused on optimizing the transplanted cells themselves. Recent studies have demonstrated that NS/PCs with spinal cord–specific patterning exhibit superior therapeutic effects compared to forebrain-type NS/PCs in SCI models.^71^^,^^72^ Based on this evidence, we established hiPSC-NS/PCs with spinal cord regional identity by modulating rostrocaudal patterning during the differentiation process.^30^ This identity was validated by the expression of HOX genes (eg, HOXB4 and HOXC4) and other spinal cord–associated markers. Regarding differentiation tendencies, hiPSC-NS/PCs used in preclinical studies for subacute SCI exhibited a predominantly neurogenic profile, with approximately 50%–70% of grafted cells differentiating into neurons.^25^^,^^73^ In contrast, to better address the pathological features of the chronic phase—such as persistent demyelination—we developed gliogenic hiPSC-NS/PCs. These cells showed enhanced potential for oligodendrocytic and astrocytic differentiation.^31^^,^^47^ The optimal balance of differentiation tendencies, however, remains to be clarified.

### Translational strategies for hiPSC-NS/PCs transplantation in chronic SCI

To facilitate clinical application of hiPSC-NS/PCs transplantation for chronic SCI, a preclinical research program has been established in Japan to develop cell transplantation therapy using clinical-grade gliogenic hiPSC-NS/PCs. This initiative—led by Nagoshi and colleagues—is supported by the Japan Agency for Medical Research and Development (AMED), a government-funded agency in Japan.

In parallel, we are also developing a rehabilitation approach based on insights gained from basic research. This includes the use of a voluntarily controlled exoskeleton, the Hybrid Assistive Limb (HAL; CYBERDYNE Inc.), in combination with body-weight-supported treadmill training.^74^ HAL detects bioelectrical signals corresponding to the user’s voluntary motor intention and assists joint movements accordingly. For patients with chronic SCI and severe motor impairment, conventional rehabilitation is often insufficient, and robot-assisted rehabilitation is expected to serve as a valuable adjunct. Although HAL alone provides limited functional recovery, its combination with hiPSC-NS/PCs transplantation is anticipated to promote reconstruction of motor function, representing a promising therapeutic strategy.^75^^,^^76^ Its clinical use is currently limited by cost, training requirements, and availability, but these challenges may be alleviated as technology and clinical adoption advance.

## Challenges for clinical translation

### Challenges in preclinical research

To date, most preclinical studies on SCI have been conducted using thoracic injury models. In fact, studies employing cervical SCI models for evaluating functional recovery account for only 2%–12% of all SCI research, and the design of many clinical trials has been based on data derived from thoracic SCI models.^77^^,^^78^ This trend is largely attributable to the challenges associated with high-level cervical injuries, including the complexity of perioperative management due to respiratory impairment and spinal shock, ethical and caregiving issues related to quadriplegia, and difficulties in quantitatively assessing upper limb function.^79^ However, in clinical practice, cervical SCI accounts for approximately 66%–88% of all SCI cases, and the resulting impairments typically involve both upper and lower limb dysfunction, leading to severe functional disability and great social burden.^2^^,^^80^ Compared with thoracic injuries, cervical SCI disrupts more complex neural circuitry, including pathways controlling skilled forelimb function and respiration.^81^ Restoring these networks requires precise axonal regeneration and accurate synaptic reintegration, making functional recovery especially challenging. By contrast, thoracic injuries mainly affect locomotor circuits of relatively lower complexity. These distinctions explain why cervical models, despite their high clinical relevance, pose unique translational challenges.^82^ Although the number of studies employing cervical SCI models remains limited, several representative studies have demonstrated encouraging outcomes. Transplantation of hESC-derived OPCs in a subacute cervical injury model promoted remyelination and improved forelimb motor and respiratory function.^83^ Moreover, transplantation of rodent fetal-derived NS/PCs combined with rehabilitation, when performed 4 weeks after a cervical contusion, enhanced corticospinal regeneration and skilled forelimb recovery.^84^ In addition, transplantation of rodent fetal-derived NS/PCs enriched with excitatory V2a interneurons facilitated donor–host circuit integration and improved respiratory motor output in a cervical contusion model.^85^

Species differences are an important consideration for translation. In rodents, skilled forelimb recovery involves multiple descending pathways, whereas in humans it depends mainly on direct corticospinal projections.^86^ Nonhuman primate studies provide an intermediate step, demonstrating long-distance corticospinal growth and broader circuit integration, thereby strengthening translational relevance.

Collectively, these considerations highlight both the progress and remaining challenges in preclinical research, emphasizing the need for further refinement of experimental models to better bridge basic findings to clinical application.

### Practical challenges for clinical translation

While preclinical research remains essential for establishing scientific and therapeutic rationale, several practical issues must also be addressed before clinical translation can be realized.

To accurately assess patient outcomes, the incorporation of objective functional evaluation metrics is essential. However, in patients with SCI, the reliability of functional assessment is significantly compromised by numerous factors. These include joint contractures, injury level and severity, baseline function, comorbid conditions, and variability in rehabilitation interventions. In particular, in cell transplantation therapies for chronic SCI, the longer time elapsed between injury and treatment compared to subacute cases increases the influence of these background factors. As a result, it is difficult to ensure homogeneity across patient cohorts, making thoughtful trial design imperative.

The expansion of noninvasive assessment methods is also a critical issue. Diffusion tensor imaging (DTI) and diffusion tensor tractography using MRI have been established as valuable tools for evaluating spinal white matter fibers after human SCI by visualizing anisotropic water diffusion.^87–89^ These techniques enable visualization of residual axonal fibers and allow for the assessment of axonal regeneration. Furthermore, q-space diffusion MRI has been applied to experimental SCI models, providing more detailed microstructural information beyond the limitations of conventional DTI—enabling enhanced assessment of axonal integrity and myelination.^90^ Moving forward, the development and application of novel noninvasive approaches to capture functional recovery—including activity changes within brain and spinal cord networks—are anticipated.^91^ Such advances are expected to facilitate the objective evaluation of not only structural but also functional improvement.

In patients with cervical SCI, who are the primary target for clinical application, the medical and caregiving burdens on both patients and their families are considerable, often presenting barriers to participation in clinical trials. Consequently, securing an adequate number of participants remains a major challenge, particularly in trials targeting chronic SCI.

Autologous hiPSCs’ approaches minimize alloimmune responses but are not yet practically feasible. They are hindered by high cost, long preparation times, and variability between manufacturing batches, making them unrealistic for urgent or large-scale clinical use. By contrast, allogeneic transplantation using HLA-homozygous donor iPSC lines offers greater scalability and immediate availability, with reduced immunological barriers.^92^ Although short-term immunosuppression may still be required, this strategy is already being implemented in Japan and is currently the most feasible pathway toward clinical translation.

Moreover, the development of cell-based products requires substantial cost and effort, and there are still no universally established criteria for evaluating efficacy. As a result, several pharmaceutical companies have withdrawn from the development of regenerative therapies for SCI.^22^ Although hiPSC-NS/PCs transplantation holds great therapeutic promise, sustained cell manufacturing, quality control, and stable supply remain critical challenges. For multicenter clinical trials, additional considerations include cryopreservation, batch-to-batch consistency, and robust shipping logistics to ensure product viability and reproducibility across sites. The reliable and safe delivery of cell-based products to clinical settings will require addressing multiple issues, including the standardization of manufacturing protocols, the establishment of scalable production technologies, and the development of a robust distribution system.

## Conclusion

Stem cell–based therapies, particularly transplantation of hiPSC-NS/PCs, have made significant progress in basic and preclinical research, primarily in the acute and subacute phases of SCI, steadily paving the way toward clinical application. In recent years, increasing attention has been directed toward therapeutic strategies for chronic SCI, where we and others have focused on establishing novel cell-based therapies using gliogenic hiPSC-NS/PCs capable of responding to the hostile microenvironment characteristic of the chronic phase.

Nonetheless, several challenges remain to be addressed before stem cell–based therapy for SCI can be translated into clinical practice. These include ensuring long-term safety, establishing objective and standardized functional outcome measures, and securing a stable supply chain for clinical-grade cell products. In particular, further advancement of basic and preclinical research specifically targeting cervical SCI will be critical for future clinical translation. Stem cell therapies based on iPSCs offer a fundamentally new treatment paradigm for SCI, which has long lacked effective interventions. Continued and rigorous accumulation of basic and preclinical evidence will be essential to support the scientific foundation for future clinical applications.

## Data Availability

The data are available from the corresponding author on reasonable request.
